# Pesticides and neurodevelopment of children in low and middle-income countries: A systematic review

**DOI:** 10.1371/journal.pone.0324375

**Published:** 2025-06-11

**Authors:** Bailey Coleman, Iqra Asad, Yi Yan Heng, Laura Menard, Faridah Hussein Were, Melissa R. Thomas, Catherine J. Karr, Megan S. McHenry

**Affiliations:** 1 Department of Pediatrics, Indiana University School of Medicine, Indianapolis, Indiana, United States of America; 2 School of Science, Indiana University Indianapolis, Indianapolis, Indiana, United States of America; 3 School of Health and Human Sciences, Indiana University Indianapolis, Indianapolis, Indiana, United States of America; 4 Ruth Lilly Medical Library, Indiana University School of Medicine, Indianapolis, Indiana, United States of America; 5 Department of Chemistry, College of Biological and Physical Sciences of the University of Nairobi, Nairobi, Kenya; 6 Department of Pediatrics, University of Washington, Seattle, Washington, United States of America; 7 Department of Environmental & Occupational Health Sciences, University of Washington, Seattle, Washington, United States of America; Suez Canal University, EGYPT

## Abstract

**Background:**

Pesticides are increasingly common in low- and middle-income countries (LMICs), where weaker regulations and multiple risk factors for poor neurodevelopment exist. Due to biological and behavioral factors, children are vulnerable to chronic pesticide exposure at a time when brain development is critical. The objective of this study is to systematically review studies assessing pesticides use with child neurodevelopment in LMICs.

**Methods:**

Using terms developed by a medical librarian, a search was performed in June 2023 across online databases, including OVID MEDLINE and EMBASE. For inclusion, studies required a measurement of pesticide exposure and neurodevelopmental outcomes using a standardized tool and study participants ≤18 years within an LMIC, as determined by World Bank criteria. Descriptive analyses were performed using extracted data, including published outcomes of significance. Results were assessed for internal validity and reported by the method of exposure measurement (biomarkers or questionnaires/region of residence).

**Results:**

A total of 31 studies spanning 11 LMICs met the inclusion criteria. An adverse association was found between pesticide exposure and at least one domain of neurodevelopment in 23 studies, including 12 studies with child-level measurements, 10 studies with maternal measurements in pregnancy, and one questionnaire-based study. Exposure to organochlorines, carbamates, chlorpyrifos, and fungicides were consistently associated with worse outcomes for neurodevelopment, specifically executive functioning, cognition, motor development, and behavior. Few studies found adverse associations with urine/serum organophosphate levels. Due to the heterogeneity of existing data, we were unable to quantify the relationship between pesticide exposure and neurodevelopment.

**Conclusions:**

While studies suggest that some domains of neurodevelopment may be negatively associated with pesticide exposure, extrapolation is limited due to the challenges in measuring pesticide exposure within these contexts and differing study designs. Several research gaps must be addressed to develop policy and regulations that protect children from potential neurodevelopmental deficits associated with pesticide exposure.

## Introduction

Pesticides have undoubtedly changed our world, allowing us to meet increasing agricultural demands and mitigate the spread of endemic insect-borne diseases. Pesticide production has increased linearly for more than half a century, with an estimated yearly consumption of 3.5 x 10^6^ tons globally in 2020 [1,2]. In their management of pest populations, pesticides have augmented the global food supply while helping extinguish endemic insect-borne disease. Studies estimate that 50% of malaria cases and 18% of child deaths related to malaria can be prevented with insecticide-treated nets, a more powerful prevention method than untreated nets [3,4]. The use of pesticides not only benefits our ability to feed and protect global populations, but also benefits the economy. Excluding external application costs, it is estimated that farmers who use pesticides gain a $6.50 return on investment for each dollar spent [5].

Despite these advantages, pesticide use is not without harm for population health. Pesticides are used extensively in the agricultural industry – where they enter the global food supply – and in residential areas, which places the applicator and the general public at risk for pesticide-related health complications [6]. The widespread use of pesticides introduces a complex dynamic of global protection compared to personal harm, where their many benefits in food production and vector-borne illness may be tempered by potential risks among chronically exposed communities. Pesticide exposures occur through inhalation, skin absorption, and/or ingestion. High exposure to acutely toxic types of pesticides can be fatal, whereas chronic exposure to pesticides with carcinogenic properties may influence cancer risk. The toxic properties of pesticide chemicals include other complications affecting every organ system in the body. Children are particularly vulnerable to the adverse effects of pesticides due to biological factors and behavioral patterns that increase their unintentional exposure to pesticides (hand-to-mouth, object-to-mouth, and playing outside barefoot). Children also consume more food and liquids per kilogram of their body weight, exacerbating their pesticide encounters. Additionally, many chemicals transfer through breast milk placenta and reside in human fat stores [7,8]. Environmental exposure to pesticides is particularly harmful in early childhood, as the human brain undergoes critical stages of neurodevelopment during this period [9].

Through direct and indirect mechanisms, pesticides can lead to complications in birth outcomes, childhood asthma, pediatric cancers, and importantly, neurocognition [10]. Multiple studies have demonstrated the negative effects of pesticide exposures on various areas of child neurodevelopment, including cognitive, motor, and behavioral deficits; however the majority of studies come from high-income countries [11–13]. Evidence from low-and middle-income countries (LMICs) is critical as these countries are disproportionately affected by pesticide exposure. The World Health Organization reported that an estimated 94% of deaths caused by environmental pollution, including the use of toxic pesticides, occur in LMICs [10]. Further, pesticides use in LMICs does not require the same rigorous standards as expected in high-income countries, due to limited funding for enforcing the regulations and educating applicators [14]. For example, the EcoSalud Project, which will appear throughout articles in this review, aimed to address the lack of government intervention on high rates of acute pesticide poisoning among smallholder farmers in Ecuador, who often use cheap, harmful pesticides and may not understand toxicity labels [15]. Pesticides that are not approved or registered for use in the United States can be lawfully manufactured on U.S. soil and exported to other countries [16]. With looser regulation in LMICs, empty pesticide containers are often left behind where children may encounter them while playing [17]. Furthermore, undernutrition and nutrient deficiencies are more common in LMICs and exacerbate the burden of pesticides on children’s health [18].

These factors, in combination with decreased access to healthcare, heighten the deleterious influence of pesticides on the health of children in LMICs [19]. However, there is no succinct resource that describes the impact they may have on child neurodevelopment within LMICs. The purpose of this study is to systematically review studies examining the effects of pesticide exposure on child neurodevelopment in LMICs and to identify gaps in the literature for future policy and practice.

## Materials and methods

We used The Preferred Reporting Items for Systematic review and Meta-Analysis (PRISMA) Protocols 2020 Checklist for this systematic review (see Supporting information). On April 28, 2020, the overarching review protocol on environment and neurodevelopmental outcomes was submitted to PROSPERO (CRD42020166245). In August 2020, an amendment was added to exclude high-income countries and split the review to three focused topic areas: pesticides, heavy metals, and air pollution. The associated review on heavy metals review has been published [20], and a review on air pollution is forthcoming.

### Search strategy

For this review, we utilized a search strategy that was developed by a medical librarian in consultation with M.S.M (see Supporting information for the search strategy). This strategy was used to search the following databases: Ovid MEDLINE, EMBASE, Cochrane Library, CINAHL, PsychInfo, Scopus, and Web of Science. The most recent database search was performed on June 22, 2023 to ensure that any articles published from the time of the initial search were included (see Supporting information for full titles from the systematic search).

### Inclusion/Exclusion criteria

The inclusion criteria was defined as follows: (A) had measurement of a pesticide exposure (either biomarker or surveys); (B) had neurodevelopment as primary outcome, measured by a standardized psychological tool; (C) setting was in a country meeting World Bank’s LMIC criteria [21,22]; (D) study population was ≤ 18 years old, including in utero; (E) followed a cross-sectional, observational cohort, quasi-experimental, or ecological study design; and (F) published after 1970. Exclusion criteria included: abstracts only, literature reviews, and case control studies involving <10 participants, as well as full-text articles not available in English. These exclusion criteria ensured the inclusion of comprehensive and validated studies with strong levels of evidence in our review.

### Selection process

Two primary reviewers (B.C. and I.A.) scanned titles and abstracts to eliminate any articles that did not meet the inclusion criteria. The full-text screening process was then conducted by a team of three students, collaborating across the three neurodevelopment and environmental systematic reviews (B.C, I.A, and Y.H.), to determine article eligibility for the current pesticides review (led by B.C.). Both the initial and full-text screenings were performed on Covidence. Each full-text article was evaluated by at least two reviewers and discussed in weekly team meetings between all three students. Any disagreements between the two initial reviewers- as determined by Covidence software- were discussed and settled with the third independent reviewer. If a consensus was not reached, the senior author (MSM), an outside reviewer, was contacted as necessary. Once the full-text screening was complete, the bibliographies of all remaining papers and literature reviews were screened to ensure that relevant articles were included. Reasons for exclusion in the full-text screening were documented for those not included within the final review.

### Data extraction

To assist in qualitative synthesis, the review team independently extracted study data into an Excel table (B.C., M.T., Y.H.). Data extraction for each study was performed by a single reviewer, and discrepancies or missing data noted in extraction were reviewed among the group to confirm details and obtain consensus. Information regarding the study design, study population (country of residence, sample size, and age at exposure), timing of neurological testing, method of measuring exposure, neurological measure, results, and limitations were noted. Studies were defined as biomarker/questionnaire and child-level/maternal when at least one component of measured exposure correlated with the appropriate classification. Data extraction was initiated in June 2020 and continued through multiple iterations to ensure accuracy and consistency, concluding in September 2024. When summarizing findings for this review, the data extraction form was frequently consulted to determine key elements that could be integrated and synthesized.

### Data synthesis and analysis

Due to differences in the exposures, methods of exposure measurement, exposure levels, and neurological tests used among the articles, a meta-analysis was not possible. Instead, a qualitative analysis was compiled. To ensure the internal validity of each article, a quality analysis was conducted for each study by two independent reviewers (B.C. and I.A.) using the National Institutes of Health Study Quality and Assessment Tools, which allowed reviewers to evaluate across differing study designs by summing the number of quality indicators to determine an overall score (see Supporting information) [23]. The number of items for scoring was split into proportional thirds, as such that cross-sectional studies were judged as good quality when scores were 9/14 and above, fair at scores of 7/14 and 8/14, and poor at 6/14 and under. Any differences in quality scores were mediated in conferring with a third reviewer (Y.H.).

## Results

### Overview of search results

**Fig 1:**
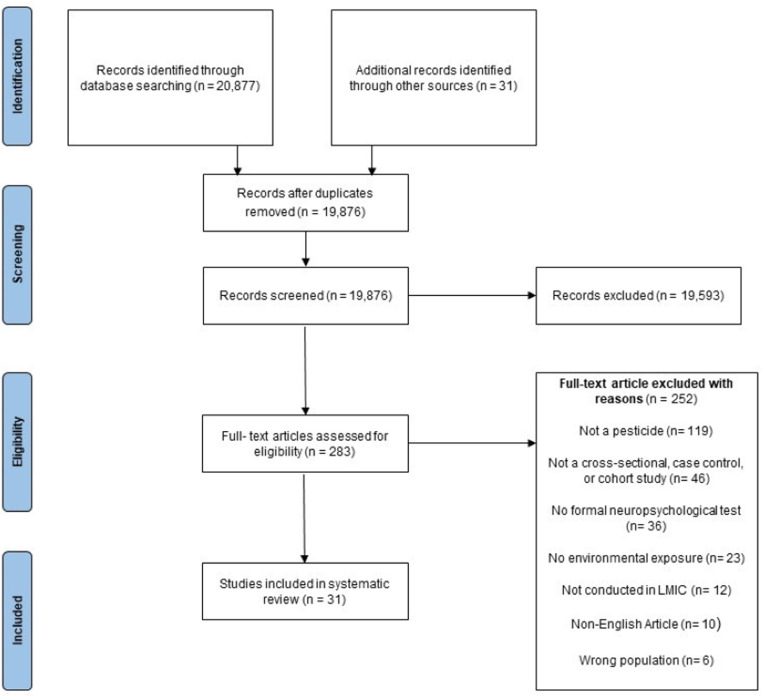
PRISMA flow diagram of included studies.

Four separate literature searches yielded a total of 20,908 articles. After combining additional records and removing duplicates, 19,876 unique articles remained. The title and abstract screening further reduced this number to 283 full-text articles, and 31 articles met the inclusion criteria and were included in this review (Fig 1).

**Fig 2:**
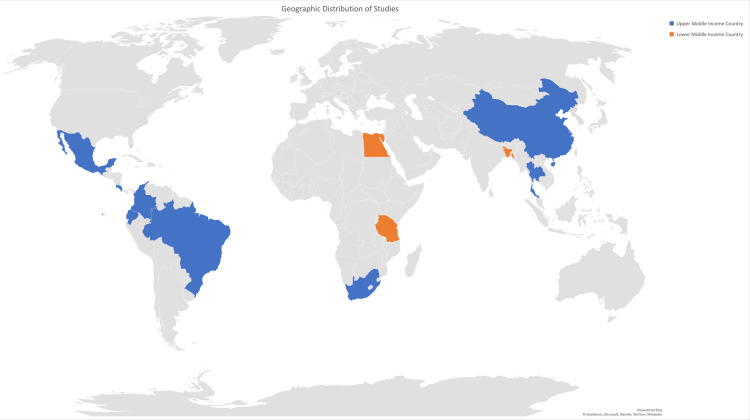
Geographic distribution of included studies. **Legend**: **Mexico** (n = 6): *Direct Observations* – Torres-Sánchez (2007), (2009), (2013), Bahena-Medina (2011), Watkins (2016); *Indirect Observations* – Guillette (1998). **Ecuador** (n = 8): *Direct Observations* – Grandjean (2006), Harari (2010), Suarez-Lopez (2017), Espinosa da Silva (2022); *Indirect Observation*s - Handal (2008), (2007) (a), (b), (c). **Costa Rica** (n = 2): *Direct Observations* – Lu (2009), Wendel de Joode (2016). **Brazil** (n = 1): *Indirect Observations* – Eckerman (2007). **Colombia** (n = 1): *Indirect Observations* – Benavides-Piracon (2022). **Egypt** (n = 3): *Direct Observations* – Abdel Rasoul (2008), Eadeh (2021), (2023). **South Africa** (n = 3): *Direct Observations* – Eskenazi (2018), An (2022); *Indirect Observations* – Chetty-Mhlanga (2021). **Tanzania** (n = 1): *Indirect Observations* – Chilipweli (2021). **Bangladesh** (n = 1): *Direct Observations* – Bliznashka (2023). **Thailand** (n = 1): *Direct Observations* – Fiedler (2015). **China** (n = 4): *Direct Observations* – Guodong (2012), Xue (2016), Zhou (2022), Chen (2022). Reprinted from GeoNames and Open Street Map under a CC BY license.

The studies were conducted in 11 different middle-income countries, including Ecuador (n = 8), Mexico (n = 6), China (n = 4), Egypt (n = 3), South Africa (n = 3), Costa Rica (n = 2), Brazil (n = 1), Thailand (n = 1), Bangladesh (n = 1), Colombia (n = 1), and Tanzania (n = 1) [21,22] (see Supporting information). The majority of studies were conducted in upper-middle income countries [24], with the exception of five studies from Egypt [25–27], Tanzania [28], and Bangladesh [29], which are classified as lower-middle income countries [30] (Fig 2). The Ecuadorian studies selected three different subset populations from the Pichincha province [31–38], however it was unclear which proportion of participants were shared among the Ecuadorian studies. For example, four studies from the EcoSalud Project used the same methods to measure cognitive development in a single subset population, but each article presented different elements of the data. Furthermore, two of the EcoSalud studies measured exposure using questionnaires, while the other two relied on the exposure levels of residence areas (i.e., high versus low) [31–34]. A different subset population resided in the Tabacundo-Cayambe region of Ecuador and was part of two different studies [35,36]. The other subset population was derived from the ESPINA study and appears in two articles in this review [37,38]. Of the six studies conducted in Mexico [39–44], four were conducted in Morelos with the same population [39–42].

The study characteristics and outcomes were subdivided based on methods of measuring the exposure: biomarkers (child and maternal measurements) and region of residence or questionnaire/survey (Tables 1–3).

**Table 1:** Study Characteristics of Included Articles using Biomarker Measurements.

Author (Year)	Extractor and Initial Date	Design	Country	Population	In Utero	Exposure	Measurement	Age at testing	Cognitive Measurement	Results	Quality
Lu (2009)	YH (June 2020)	Cross-sectional	Costa Rica	n = 17 children whose parents worked in coffee plantation n = 18 children whose parents worked in their own conventional coffee farms	No	Organophosphates and pyrethroids (PYR); herbicides (5-chloro-1-isopropyl-3-hydroxytriazole; 2-isopropyl-6-methyl-4-pyrimidinol); 3-Phenoxybenzoic acid (3-PBA); and 3,5,6-trichloro-2-pyridinol (TCPy); 2,4-Dichlorophenoxyacetic acid	Urine	4-10 years	Behavioral Assessment and Research System (BARS); a figure-drawing task; a long-term memory test	Variables outside of pesticide exposure (e.g., family socioeconomic status) were associated with greater impacts on cognitive development. Children whose parents worked in a small farm performed better in BARS and the figure drawing tests than did children whose parents were working in a plantation, but there were no significant differences in these two groups.	Good
Wendel de Joode (2016)	BC (July 2020)	Cross-sectional	Costa Rica	n = 140 children aged 6–9 years	No	PYR, mancozeb, TCPy, Chlorpyrifos (CPF), 3-PBA	Urine	6-9 years of age	Wechsler Intelligence Scale for Children (WISC)- IV; Conner’s Parent Rating Scale-Revised Short Version; Lanthony Desaturated D-15; Rey-Osterrieth Complex Figure; Children’s Auditory Verbal learning Test- 2nd edition; Frostig Developmental Test of Visual perception- 2nd edition; Wide Range Assessment of Visual Motor Ability; Reaction Time Test	Greater TCPy concentrations were correlated with decreased working memory in boys (n = 59) [CI: −14.4 to −0.7]; worse visual motor coordination [CI: −2.7 to −0.1]; increased rates of inattention [CI: 1.6 to 22.9], oppositional disorders [CI 1.0 to 16.0], and ADHD [CI: 1.8 to 28.6]; and poorer color discrimination [CI: 1.6 to 30.3]. Lower verbal learning scores were noted [CI: −12.7 to – 1.3] in those with higher ethylenethiourea levels. Processing speeds were slower in those with increased 3-PBA levels, especially in girls [CI: −16.1 to −1.4].	Good
Grandjean (2006)^*^	YH (June 2020)	Cross-sectional	Ecuador	n = 37 exposed to pesticides n = 35 controls	Both in utero and postnatal exposures measured	Pesticides of which organophosphates are the primary	Questionnaire, acetylcholinesterase, urine	6-8 years of age	Santa Ana Form Board; WISC-Revised Digit Spans forward; Stanford-Binet Copying Test; Catsys force plate (simple reaction time)	Exposed children had lower scores than control children in copying designs. Significant associations were noted between longer reaction time and postnatal organophosphate exposure [P = 0.011].	Good
Harari (2010)^*^	YH (June 2020)	Cross-sectional	Ecuador	n = 83 children aged 6–8 years	Both in utero and postnatal exposures measured	Pesticides of which organophosphates are the primary use	Questionnaire, acetylcholinesterase, urine	6-8 years of age	Finger Tapping Task; Santa Ana Form Board; Conners’ Kiddie Continuous Performance Test; Copying Test of the Stanford Binet; Raven’s Colored Progressive Matrices; WISC-Revised; digit span test; Stanford Binet Memory for Sentences and Digit String tests	Prenatal exposure was the only exposure associated with significant developmental delays after covariate adjustment. The strongest correlation between delays and maternal exposures were noted in visual memory [CI: 1.02 to 42.93], visuospatial performance [CI: 0.2 to 1.0], motor speed [CI: –12.5 to –1.6], and motor coordination [CI: 1.3 to 27.62].	Good
Suarez-Lopez (2017)^*^	YH (June 2020)	Cohort	Ecuador	n = 308 children aged 4–9 years	N/A	Pesticides, insecticides, herbicides, diethyldithiocarbamate fungicides and organophosphate insecticides (primary)	Time after Mother’s Day harvest, acetylcholinesterase	4-9 years	Developmental NEuroPSYchological Assessment -II	Children examined closer in date to the Mother’s Day had lower neurobehavioral scores than children examined later in domains of: Attention/Inhibitory Control [CI: 0.10 to 0.65], Visuospatial Processing [CI: 0.25 to 0.95], and Sensorimotor [CI: 0.10 to 0.77], and total neurobehavior [CI: 0.08 to 0.44].	Good
Espinosa da Silva (2022)^*^	MT (October 2023)	Cohort	Ecuador	n = 842 children aged 11–17 years	No	Organophosphates, insecticides, others possible as not directly measured or noted	Time after Mother’s Day harvest; acetylcholinesterase	11-17 years	Developmental NEuroPSYchological Assessment-II	Per 10 days following harvest, attention and language domains decreased by 0.22 and 0.19 points, respectively. In follow-up, attention and visuospatial processing domains maintained a direct association with time after the harvest [CI: 0.04 to 0.34; −0.29 and −0.09, respectively].	Good
Torres-Sánchez (2007)^*^	BC (July 2020)	Cohort	Mexico	n = 244 children from pregnancy to 12 months	Yes	Dichloro-diphenyl-dichloroethylene (DDE)	Maternal serum	1, 3, 6, and 12 months of age	Bayley’s Scales of Infant Development (BSID)–III: Psychomotor Developmental Index (PDI) and Mental Development Index (MDI)	No correlations were documented between DDE and MDI, but there were significant reductions in PDI for each doubled increase in DDE level during the first trimester of pregnancy [P = 0.02].	Good
Torres-Sánchez (2009)^*^	BC (July 2020)	Cohort	Mexico	n = 270 children from pregnancy to 30 months	Yes	DDE	Maternal serum	12, 18, 24 and 30 months of age	BSID-II	Correlations between prenatal DDE exposure and neurodevelopment deficits are no longer significant at 12 months of age.	Good
Bahena-Medina (2011)^*^	BC (July 2020)	Cohort	Mexico	n = 265 children from pregnancy to 1 month (+/- 7 days)	Yes	DDE	Maternal serum	1 month (+/- 7 days)	BSID; Graham-Rosenblith Scale; Brazelton Scale reflexes	Increases in neurological soft signs and decreases in psychomotor and mental development were documented for children prenatally exposed to DDE, but the findings did not meet statistical significance.	Good
Torres-Sánchez (2013)^*^	BC (July 2020)	Cohort	Mexico	n = 203 children at ages 42, 48, 54, and 60 months	Yes	DDE	Maternal serum	42, 48, 54, and 60 months	McCarthy Scales of Children’s Abilities	For each doubling of DDE, there was a significant reduction in points associated with following areas: general cognitive index [–1.37], quantitative [–0.88], verbal [–0.84], and memory skills [–0.80].	Good
Watkins (2016)	BC (July 2020)	Cohort	Mexico	n = 187 from pregnancy to 36 months	Yes	PYR	Maternal urine	24 and 36 months	BSID-II: PDI and MDI	No correlations were noted for MDI at 36 months or with PDI scores at any of the points in time.	Good
Abdel Rasoul (2008)	BC (July 2020)	Cross-sectional	Egypt	n = 30 children aged 9–15 years and occupationally exposed n = 20 children aged 16–19 years	No	Organophosphates (various forms of CPF), PYR (or less potent carboxylate)	Questionnaire, acetylcholinesterase	9-19 years	Wechsler Adult Intelligence Scale; Eysenck Personality Questionnaire	Overall, those that applied pesticides had decreased neurobehavioral scores in both the younger and older groups. Further analysis demonstrated a dose-effect correlation between exposure to pesticides and cognitive deficits in 3–6 subtests among children occupationally exposed to pesticides.	Good
Eadeh (2021)^*^	BC (April 2022)	Cohort	Egypt	n = 242 males aged 12–18 (age at recruitment)	No	CPF, TCPy	Urine	12-21 years	BARS	Mean TCPy exposure levels were adversely associated with scores on digit span reverse, match to sample, serial digit learning, and tapping, alternating [all P < 0.05].	Good
Eadeh (2023)^*^	MT (October 2023)	Cohort	Egypt	n = 226 males aged 12–18 (age at recruitment)	No	CPF, PYR, alpha-cypermethrin, lambda-cyhalothrin, TCPy, 3-PBA, 2,2-dichlorovinyl-2,2-dimethyl-1-cyclopropane carboxylic acid (DCCA)	Urine	12-21 years	ADHD-Rating Scale-IV	Out of the measured biomarkers, only cis-DCCA was associated with greater symptoms of ADHD [CI: 1.97 to 12.39].	Good
Eskenazi (2018)^*^	YH (June 2020)	Cohort	South Africa	n = 752 children from birth-2 years	Yes	Dichloro-diphenyl-trichloroethane (DDT), DDE, DCCA, PYR	Maternal blood and urine	Assessed at 1 year of age and again at 2 years of age	BSID–III: Cognitive, Language, and Social-Emotional subtests.	No significant associations were found between cognitive delays and elevated DDT/DDE levels. Each 10-fold increase was associated with a decrease in social-emotional scores at 1 year of age in: trans-DCCA [CI: −0.96 to −0.02], cis-DCCA [CI: −1.25 to 0.15] and 3-PBA [CI: −1.23 to −0.06].	Good
An (2022)^*^	MT (October 2023)	Cohort	South Africa	n = 683 mother-child pairs, children aged 0–2 years	Yes	PYR; 3-PBA; DDE, DDT; DCCA	Urine	2 years	Child Behavior Checklist	Per 10-fold increase in DDT or DDE concentrations for maternal serum, there were three corresponding relationships: 0.24-point increase on child withdrawn behavioral score; 1.67-point increase in oppositional-defiant behavior;-1.72 increase in ADHD-related problems. Urinary concentrations of 3-PBA and cis-DCCA had a level of correlation with externalizing behaviors and affective disorders [RR = 1.30 and 1.25, respectively].	Good
Bliznashka (2023)	MT (October 2023)	Cohort	Bangladesh	n = 284 mother-child pairs, children aged 0–40 months	Yes	2,4- Dichlorophenoxyacetic acid; TCPy; 4-nitrophenol; malathion dicarboxylic acid; 2-isopropyl-4-methyl-6-hydro-xypyrimidine (IMPy); 4-fluoro-3-PBA; 3-PBA; trans-DCCA	Urine	20-40 months	BSID-III	A systematic review component found no correlations between 3-PBA levels in pregnancy with child development, supported by the Bangladesh cohort. 4-nitrophel also yielded no correlation with child development measures. Concentrations of IMPy and TCPy were inversely correlated with motor development [CI: −1.23 to −0.09] and cognitive development [CI: −0.04 to 0.01], respectively.	Good
Guodong (2012)	BC (July 2020)	Cross-sectional	China	n = 301 children aged 23–25 months	No	Organophosphates	Urine	23-25 months	Gesell Developmental Schedules	No significant associations between developmental measures and organophosphate levels were noted.	Good
Xue (2013)	BC (July 2020)	Cross-sectional	China	n = 497 children from pregnancy to 12 months	Yes	Synthetic PYR pesticides	Urine	12 months	Development Screen Test: MDI	A significant adverse correlation was found between PYR exposure and neural and mental development [P < 0.05].	Good
Zhou (2022)	MT (October 2023)	Cross-sectional	China	n = 673 children aged 1–6 years	No	CPF	Urine	1-6 years	Diagnostic and Statistical Manual of Mental Disorders	Out of 651 who were assessed, 45 were categorized as positive for ADHD (6.9%). Accordingly, CPF had a direct relationship with greater ADHD risk [P < 0.05].	Good
Chen (2022)	MT (October 2023)	Cohort	China	n = 327 children from pregnancy to 2 years	Yes	PYR; DCCA; 3-PBA	Urine	2 years	BSID-III	Compared to children not exposed to PYR with DCCA, children who were exposed had a 22% higher risk of language development delay [P < 0.001]; receptive communication domains were also affected by 3-PBA in children exposed [P < 0.006]. Language and communication scores, overall, were adversely correlated with PYR exposure [P = 0.042, 0.010, respectively].	Good
Fiedler (2015)	YH (June 2020)	Cross-sectional	Thailand	n = 24 rice farming (exposure) n = 20 aquafarm (control) children aged 6–8 years	No	Organophosphates, PYR, CPF, TCPy. 3-PBA, DCCA	Urinary metabolites	6-8 year olds (3 different sessions were conducted for trial season)	Behavioral Assessment and Research System (BARS)	Concentrations of dialkylphosphates (low season P = 0.002; high season P = 0.006) and TCPy were higher in rice farm children than aquafarm children prior to covariate adjustment. No significant differences were noted between groups or season for PYR metabolites, but memory and motor tests showed better scores for children exposed to DCCA and 3-PBA.	Good

* These studies featured the same population, general study design, and overall results, but differed in the way they analyzed and reported data.

Abbreviations: PYR: pyrethroids; 3-PBA: 3-Phenoxybenzoic acid; TCPy: 3,5,6-trichloro-2-pyridinol; BARS: Behavioral Assessment and Research System; CPF: Chlorpyrifos; WISC: Wechsler Intelligence Scale for Children; DDE: dichloro-diphenyl-dichloroethylene; BSID: Bayley’s Scales of Infant Development; PDI: Psychomotor Developmental Index; MDI: Mental Developmental Index; DCCA: 2,2-(dichloro)-2-dimethylvinylcyclopropane carboxylic acid; DDT: dichloro-diphenyl-trichloroethane; IMPy: 2-isopropyl-4-methyl-6-hydroxypyrimidine.

**Table 2:** Study Characteristics of Included Articles using Questionnaire Assessments.

Author (Year)	Extractor and Initial Date	Design	Country	Population	In Utero	Exposure	Measurement	Age at testing	Cognitive Measurement	Results	Quality
Eckerman (2007)	YH (June 2020)	Cross-sectional	Brazil	Rural n = 38, worked on family farm Urban n = 28	No	Pesticides	Questionnaire	10-18 years (analysis subgroups for ages 10–11, 12–13, 14–15)	Behavioral Assessment and Research System	Strongest, most consistent adverse correlations between pesticide exposure and neurodevelopment were documented in tapping [P = 0.08]; digit span [P = 0.07], and selective attention [P = 0.07] scores. The correlation was particularly strong in the youngest participants for a majority of the subtests.	Fair
Handal (2007a)^*^	YH (June 2020)	Cross-sectional	Ecuador	n = 142 children aged 24–61 months	Both in utero and postnatal exposures measured	Organophosphates, carbamates	Questionnaire	24-61 months	ASQ; Visual Motor Integration Test	Higher developmental scores were significantly correlated with current maternal employment in the flower industry.	Good
Handal (2007b)^*^	YH (June 2020)	Cross-sectional	Ecuador	n = 154 children in high-exposure communities n = 129 children in low-exposure community	No	Organophosphates, carbamates	Community of residence	Two groups: 3–23 months, 24–61 months	Ages and Stages Questionnaire (ASQ)	Children aged 3–23 months old scored lower in gross motor skills (30.1%). Children aged 48–61 months old scored lower in problem-solving skills (73.4%) and fine motor skills (28.1%).	Good
Handal (2007c)^*^	YH (June 2020)	Cross-sectional	Ecuador	n = 154 children in high-exposure communities n = 129 children in low-exposure community	No	Organophosphates, carbamates	Community of residence	Two groups: 3–23 months, 24–61 months	ASQ	Low gross motor [P = 0.002] and socio-individual scores [P = 0.02] were significantly associated with exposure among children aged 3–23 months residing in high-exposure communities.	Good
Handal (2008)^*^	BC (July 2020)	Cross-sectional	Ecuador	n = 121 children aged 3–23 months	Yes	Organophosphates, carbamates	Questionnaire	Between 3–23 months (one screening was conducted)	ASQ: visual acuity and precision	Once adjusted for confounding variables, maternal employment during pregnancy was associated with lower communication scores [95% CI: −16% to 0.5%], fine motor skills [CI: −22%, to 5%], and visual acuity [CI: 1.1 to 20]. Use of pesticides at home during pregnancy showed a positive correlation with gross motor skills.	Good
Benavides-Piracon (2022)	MT (October 2023)	Cross-sectional	Colombia	n = 232 aged 7–10 years	Yes	Organophosphates; synthetic pyrethroids insecticides; and fungicides	Questionnaire	7-10 years	Wechsler Intelligence Scale for Children-IV	Prenatal exposure to pesticides adversely affected overall IQ [CI: −7.14 to 0.84], where postnatal and prenatal exposure affected verbal comprehension [CI: −6.97 to 0.21; −8.36 to 1.51, respectively]. Overall, pesticide exposure adversely affected working memory [CI: −6.65 to −0.28], but not perceptual reasoning.	Good
Guillette (1998)	BC (July 2020)	Cross-sectional	Mexico	n = 33 exposed to elevated levels of pesticides n = 17 unexposed	No	Organophosphates, organochlorines, pyrethroids	Region of residence	48-62 months	Rapid Assessment Tool for Preschool Children	The exposed children demonstrated decreases in stamina, measured by jumping [P = 0.05], gross and fine eye-hand coordination [P = 0.009], 30-minute memory [P = 0.027], and the ability to draw a person [P < 0.0001].	Good
Chetty-Mhlanga (2021)	BC (April 2022)	Cross-sectional	South Africa	n = 1001 children aged 9–16	No	Organophosphates, organochlorines, others possible as not directly measured or noted	Questionnaire	9-16 years	Cambridge Automated NeuroPsychological Battery	The following pairs suggest that increased pesticide exposure was associated with lower neurocognitive scores: increased pesticide-related farm activities and lower multi-tasking accuracy scores [P = 0.03]; eating fruit directly from vineyard/orchard and both lower motor screening speed and rapid visual processing accuracy scores [both P = 0.02); picking crops off field instead of not picking crops from field and both lower strategy in spatial working memory and lower paired associated learning [P = 0.03; 0.02 respectively].	Good
Chilipweli (2021)	BC (April 2022)	Cross-sectional	Tanzania	n = 286 mother-child pairs, children aged 0–6 years	Both in utero and postnatal exposures measured	Organophosphates, pyrethroids, carbamates, glycine derivative, phthalic acid diamide, insecticides	Questionnaire	0-6 years	Malawi Child Development Tool	Children were more likely to have neurodevelopmental effects if their mothers worked while pregnant [P = 0.011], did not receive proper pesticide training [P = 0.007], were exposed for more than a year [P = 0.003], drank alcohol during pregnancy [P = 0.035]; and if the children were underweight [P = 0.01] or they lived within 5 km of a farm [P = 0.000]. Children’s weight status appeared to be an effect modifier [AOR = 7.8(1.29–36.3)] when assessed with working during pregnancy.	Good

* These studies featured the same population, general study design, and overall results, but differed in the way they analyzed and reported data.

Abbreviations: ASQ: Ages and Stages Questionnaire.

**Table 3:** Exposure and Cognition Assessments of Included Articles.

Organophosphates						Organochlorines	Chlorpyrifos	Pyrethroid Metabolites			Herbicides	Fungicides
		Dialkyl Phosphate Metabolites (six common)	Acetylcholinesterase	5-chloro-1-isopropyl-3-hydroxytriazole	2-Isopropyl-6-methyl-4-pyrimidinol	Dichloro-diphenyl-trichloroethane/ dichloro-diphenyl-dichloroethylene	3,5,6-trichloro-2-pyridnol	3-phenoxy benzoic acid	4-fluoro-3-phenoxybenzoic acid	cis- and trans-3-(2,2-dichlorovinyl)-2,2-dimethylcyclopropane-1-carboxylic acid	2,4-Dichlorophenoxyacetic acid	Ethylene thiourea
Executive Functioning Processing Speed	Reaction Time Test						O^*^	**X** ^**^				O
	Simple Reaction Time	**X**	O									
	Rapid Visual Processing	**P**										
Executive Functioning General	Rey-Osterrieth Complex Figure						**X**	**X**				O
	Copying Test of Stanford Binet	**X** O	**X**									
Executive Functioning Memory	Stanford Binet Memory for Sentences and Digit String Tests	**X**	**X**									
	Children’s Auditory Verbal Learning Test 2nd Edition						**X**	O				**X**
Executive Functioning Attention	Conner’s Kiddie Continuous Performance Test	O	O									
Cognitive Assessments	Bayley Scales—Mental Developmental Index, 2nd or 3rd ed.					O O** X**		O** X**	O	O		
	Bayley Scales—Cognitive Index, 2nd or 3rd ed.				**X**	O	**X**					
	Bayley Scales—Language Index, 3rd ed.					O		**X**	O	**X**		
	Bayley Scales—Social-Emotional Index, 3rd ed.							**X**		**X**		
	Wechsler Intelligence Scale for Children—IV	**X** (specific metabolite unknown)					**X**	**X**				
	Wechsler Intelligence Scale for Children—Revised	**X X**	**X **O									
	Wechsler Adult Intelligence Scale	**X**	**X**									
	Raven’s Colored Matrices	**X**	**X**									
	Behavioral Assessment and Research System	O O **X P**		O	O		O O **X P**	O **P**		O **P**	O	
	McCarthy Scales of Children’s Abilities					**X**						
	Gesell Developmental Schedules	O										
	Developmental Neuro-PSYchological Assessment-II	**X** **X** (specific metabolite unknown)	**X X**									**X**
	Development Screen Test							**X**		**X**		
	Cambridge Automated Neuro-Psychological Battery	**X**										
General Motor/ Psychomotor	Santa Ana Foam Board	**X **O	**X **O									
	Finger Tapping Task	**X**	**X**									
	Bayley Scales- Psychomotor Index, 3rd ed.				**X**	**X X**	**X**	O	O	O		
	Eye-hand Coordination subtest of the Frostig Developmental Test of Visual Perception						**X**	**X**				
	Wide Range Assessment of Visual Motor Ability						**X**	**X**				
	Ages and Stages Questionnaire	**P X X X**										
Behavior	Conner’s Parent Rating Scale						**X**					
	Attention Deficit Hyperactivity Disorder assessments						**X**			**X**		
	Child Behavior Checklist					**X**		**X**		**X**		
	Graham-Rosenblith Scale					**X**						
	Brazelton Neonatal Behavioral Assessment Scale					**X**						

* One symbol of X, O, or P represents a single study. Two of these symbols show the results of two separate studies.

** In a negative correlation X, children perform worse as levels of pesticide exposure increases. In a positive correlation P, children perform better as pesticide exposure increases.

Key: X = negative correlation between the pesticide and given outcome; O = no association observed between pesticide and given outcome; P = positive association between pesticide and given outcome. Blue squares represent studies of child-level biomarkers. Orange squares represent studies of maternal-level biomarkers. Green squares represent studies of dual detection methods. Yellow squares represent non-biomarker studies, e.g., questionnaires, region of residence, etc.

### Biological indicators

A total of 22 studies directly measured pesticide exposure using biological indicators, including child-level measurements (n = 13) and maternal-level measurements (n = 9).

#### Child-level measurements.

Child-level measurements included testing for urinary metabolites (n = 8), serum analysis of acetylcholinesterase (n = 3), and both (n = 2) across Costa Rica, China, Thailand, Egypt, and Ecuador.

*Studies assessing exposure using urine specimen analysis:* Eight studies focused on specific and nonspecific urinary metabolites as measures of pesticide exposure in children of various ages from 2−21 years old [26,27,45–50]. These were conducted in China (n = 3), Costa Rica (n = 2), Egypt (n = 2), and Thailand (n = 1), and all were judged as good quality. Three studies reported null findings on the association of pesticides with child neurodevelopment [45–47]. One of these studies, based in Thailand, observed an opposite effect, where increased exposure to dialkylphosphates showed improvements in accuracy and latency response times among children (*P* = 0.05; 0.008, respectively), as well as improved motor speed and learning associated with 3,5,6-trichloro-2-pyridinol (TCPy) (*P* = 0.3; 0.05, respectively) [45]. Pyrethroids were also positively associated with improved latency of response (*P *= 0.04). In this group, children recruited from rice and aquaculture farming regions showed higher levels of TCPy concentrations in the low pesticides use season compared to the high pesticides use season [45].

Five studies found statistically significant adverse associations between pesticide exposure and neurodevelopment in Costa Rica, Egypt, and China [26,27,48–50]. In Costa Rica, researchers reported a relationship between exposure and deficits in neurodevelopment among 140 children aged 6−9 years old [48]. The effect on neurodevelopment varied based on the type of pesticide and sex of the subject. Elevated TCPy concentrations were associated with increased parent-reported cognitive problems/inattention (adjusted odds ratio [aOR] = 5.8; 95% CI [1.6, 22.9]), oppositional disorders (aOR = 3.9; 95% CI [1.0, 16.0]), and ADHD-related problems (aOR = 6.8; 95% CI [1.8, 28.6]). Higher TCPy concentrations were also associated with decreased visual motor coordination (β = − 1.4; 95% CI [- 2.7,- 0.1]), ability to discriminate colors (aOR = 6.6; 95% CI [1.6, 30.3]), and working memory in boys (n = 59) (β = − 7.5; 95% CI [- 14.4, −0.7]). Elevated 3-Phenoxybenzoic acid (3-PBA) levels were identified with lower processing speed scores as particularly noted in girls (β = − 8.8; 95% CI [- 16.1, −1.4]). Elevated ethylenethiourea (ETU) concentrations were also associated with decreased verbal learning outcomes (β = −7.0; 95% CI [−12.7, −1.3]) [48].

A second set of studies in Egypt found significant effects on neurodevelopment in 12–21-year-old boys who were occupationally exposed to pesticides [26]. At enrollment, all the boys were between the ages of 12−18 and therefore met the inclusion criteria to be included in this review. Urine samples were collected >13 times over six years to determine a mean TCPy exposure level for each participant. Researchers found a negative correlation (*P* < 0.05) between TCPy exposure levels and digit span reverse (β = −0.025), match to sample (β = −0.005), serial digit learning (β = −0.049), and tapping alternating (β = −0.041) [26]. A follow-up study of behavioral outcomes showed an association with ADHD-related symptoms and cis-3-(2,2- dichlorovinyl)-2,2-dimethylcyclopropane carboxylic acid (cis-DCCA) (odds ration [OR] = 3.28, 95% CI [1.30, 8.26]), as well as a non-statistically significant positive relationship between TCPy and ADHD-related symptoms when controlling for cis-DCCA [27].

Among children aged 1–6 years in China evaluated for ADHD-related behavioral problems, urinary chlorpyrifos showed a direct effect for those at-risk for ADHD (*P* < 0.05) [49]. Among 327 children in China observed from birth, it was found that infants who were exposed to pyrethroids daily had a four-fold increased risk of language development delay than infants who were not exposed [50]. Pyrethroid exposure in infancy also was associated with lower receptive communication and language development scores in toddlers (*P* < 0.042; 0.010, respectively). Increasing concentrations of 3-PBA had an adverse effect on receptive communication for toddlers (*P *< 0.001), but not in overall language development [50].

*Studies assessing exposure using serum measurement of acetylcholinesterase:* Three studies measured serum levels of acetylcholinesterase [25,37,38]. These studies took place in Ecuador (n = 2) and Egypt (n = 1), and all were judged as good quality. The Ecuadorian studies appeared to use similar subsets of the population. One Ecuadorian study analyzed the effect of an annual flower harvest on neurobehavioral performance in children aged 4–9 years [37]. Children tested sooner after the harvest performed worse in three developmental outcome measures and in total neurobehavior, with a mean score difference of 0.26 for each 10.8 days after the harvest (95% CI [0.08, 0.44]) [37]. The most recent Ecuadorian study showed that child evaluated within ten days after the harvest had lower scores of attention/inhibitory behavior and language comprehension [38]. Longitudinal follow-up scores also showed a positive association with attention and visuospatial processing following the harvest day (β = 0.20; 95% CI [0.05, 0.35], p < 0.05; β= − 0.19, 95% CI [−0.29, −0.09], respectively) [38].

These results were similar to those of the study in Egypt, which utilized participant interviews for children 9−15 years old alongside serum acetylcholinesterase measurements [25]. Acetylcholinesterase levels were lower in the applicator group exposed to organophosphates and Pyrethrinsor (mean = 239:8; S.D. = 60.0 IU/L), a less potent carboxylate, than the control group (mean = 239:8; S.D. = 60.0 IU/L) (t = 3.6; *P *< 0.05). The control group scored significantly better on all neurobehavioral tests compared to the applicator group (*P* = < 0.001–0.04) [25].

*Dual detection methods:* Two studies recorded both urinary metabolites and serum acetylcholinesterase activity in 6–8 year-old children to quantify current pesticide exposure, while employing a maternal interview to account for prenatal exposures [35,36]. The primary pesticide class of focus for both studies was organophosphates. Both were conducted in the Tabacundo-Cayambe region of Ecuador, and the study population appears to be the same within the two studies. Both studies were judged as good quality. The initial study found that prenatal pesticide exposure was associated with decreased Stanford-Binet copying scores (designs 13–20, p = 0.2) (all designs, *P* = 0.3), while current pesticide exposure- as demonstrated by urinary metabolites- increased simple reaction times (*P* = 0.011) [36]. No associations were found between any test measures and acetylcholine esterase levels [36]. The second study included an expanded consideration of various confounding variables, including cultural factors, to their study design [35]. In this study, only maternally-reported pesticide exposure in the prenatal period appeared to adversely alter neurodevelopment in the following areas: visuospatial performance (β = 0.5; 95% CI [0.2, 1.0]), visual memory (β = 6.62; 95% CI [1.02, 42.93]), motor speed (β = –7.1; 95% CI [–12.5,–1.6]), and motor coordination (β = 5.32; (95% CI [1.03, 27.62]) [35].

#### Maternal-level measurements.

Maternal-level measurement methods consisted of analyzing maternal serum (n = 4), antenatal urine (n = 3), and both (n = 2). These studies evaluated neurodevelopment in the children of these mothers from birth up to 60 months of age and took place in Mexico, China, and South Africa.

*Studies using pesticide measures in prenatal urine:* Three studies, taking place in China (n = 1), Bangladesh (n = 1), and Mexico (n = 1), measured pyrethroid metabolites in prenatal maternal urine and evaluated its effects on motor and psychomotor indices in children less than three years of age [29,43,51]. All studies were judged as good quality. One study reported an adverse association between pyrethroid exposure and development in young children (β = − 0.152, *P *< 0.05) [51]. The trimester or timing of prenatal urine collection was not stated [51]. A second study categorized 187 maternal-child dyads into low, medium, and high pyrethroid metabolite levels (measured during the third trimester of pregnancy) and found a trend towards lower mental developmental index (MDI) scores for those in the high and medium categories at 24 months, although the scores were not statistically significant (*P* = 0.07) [43]. No further differences in MDI were found at 36 months or at 24 and 36 months for psychomotor developmental index (PDI) scores [43]. The third study found little correlation between 3-PBA and 4-nitrophel levels with child development outcomes among 284 mother-child pairs in Bangladesh. An inverse correlation was observed with 2-isopropyl-4-methyl-6-hydro-xypyrimidine (IMPy) and language development scores when unadjusted (mean difference [MD]: − 0.96; 95% CI [−1.74, −0.18]), and motor development scores when adjusted for cofounders (aMD= − 0.66; 95% CI [−1.23, −0.09]), as well as a small level of correlation between TCPy and cognitive development scores (aMD= − 0.02; 95% CI [−0.04, −0.01]) [29].

*Studies using pesticide measures in maternal serum:* Pesticide levels were quantified utilizing maternal serum screening in four studies using the same cohort in Morelos, Mexico; all were judged as good quality [39–42]. All four studies measured dichloro-diphenyl-dichloroethylene (DDE), a specific breakdown product of the organochlorine dichloro-diphenyl-trichloroethane (DDT), in each trimester and utilized Bayley’s Scales of Infant Development. The government selected DDT to treat malaria in this area until 1998. Three of these studies analyzed DDE levels before pregnancy [39–41]; while the studies shared the same population, they analyzed the neurodevelopmental outcomes at varying ages. The initial study was conducted in children from birth to 12 months and found a 0.52 point reduction in PDI scores (95% CI [–0.96 to –0.075], *P* = 0.02) for every double increase in DDE levels during the first trimester of pregnancy [40]. This association was not seen in other trimesters of pregnancy or for MDI scores [40]. Two following studies, which evaluated neurodevelopment at one month of age and 12–30 months of age, respectively, found no statistically significant changes in neurodevelopment, neurological soft signs, or reflexes [39,42]. The same population was tested again between 42–60 months using McCarthy’s Scales of Children’s Abilities [41]. These results indicated an association between a doubling of DDE levels during the third trimester of pregnancy and changes of –1.37 in the general cognitive index, as well as –0.88 in quantitative, –0.84 verbal, and –0.80 memory elements (*P* < 0.05) [41].

*Dual detection methods:* Two studies of the same cohort utilized two methods of pesticide exposure among mothers to examine their potential associations with cognitive, behavioral, and motor development in South Africa [52,53]. Both studies were judged as good quality. Maternal blood and urine sampling were used to determine DDT/DDE levels and pyrethroid levels, respectively. Some samples were taken antenatally, while others were collected during the immediate postnatal period (prior to hospital discharge). While DDT and DDE levels had no association with neurodevelopment at one year of age, significantly worse language scores were associated with every 10-fold increase for the three following pyrethroid metabolites: cis-DCCA, (β = − 0.70, 95% CI [−1.25, − 0.15]); trans-DCCA, (β = − 0.49, 95% CI [− 0.96, − 0.02]); and 3-PBA, (β = − 0.65, 95% CI [−1.23, − 0.006]) [52]. For behavioral outcomes at two years of age, every 10-fold increase in DDT and DDE concentrations corresponded with point increases in child withdrawn behavior (95% CI [0.00, 0.49]; [− 0.06, 0.53], respectively), opposition-defiant behavior (95% CI [1.01, 1.67]; [1.01, 1.91], respectively), and ADHD-related behavioral problems (95% CI [0.98, 1.72], DDE only) [53].

At two years of age, every 10-fold increase in maternal cis-(2,2-dibromovinyl)-2,2-dimethyl-cyclopropane-1-carboxylic acid (cis-DBCA) was related to decreases in Language Composite (β = −1.90, 95% CI [− 3.67, − 0.14]) and Expressive Communication scores (β = − 0.41, 95% CI [− 0.81, − 0.01]) [52]. Furthermore, girls aged two years had significantly lower scores than boys of the same age in motor scores associated with pyrethroid exposures [52]. Externalizing behavioral problems in children at two years of age were positively correlated with every 10-fold increase of cis-DBCA and 3-PBA (95% CI [1.05, 1.62]; [1.03, 1.78], respectively). A relationship between cis-DBCA and affective disorders was also observed per 10-fold increase (95% CI [0.99, 1.56]), though this association was less precise [53].

### Studies assessing exposure through surveys/Location

Region of residence (n = 3) and questionnaires/interviews (n = 6) operated as the main method of indirectly measuring pesticide exposure in nine studies. These studies took place in Ecuador (n = 4), Mexico (n = 1), South Africa (n = 1), Tanzania (n = 1), Colombia (n = 1) and Brazil (n = 1) in children ranging from birth to 18 years of age.

#### Region of residence.

In three studies taking place in Ecuador (n = 2) and Mexico (n = 1), the subjects’ region of residence served as the primary quantifier of pesticide exposure, where there is documented use of organophosphates, agricultural chemicals, and other pesticides. The Ecuadorian studies featured the same population, study design, and overall results, but differed in the way they analyzed and reported data [32,33]. The Ecuadorian studies were both judged as good quality, as well as the Mexico-based study [44]. One study reported lower gross motor (*P* = 0.002) and social scores (*P* = 0.02) for children aged 3–23 months who are living in high-exposure regions compared to those residing in low-exposure regions [32]. Possible associations between lower developmental scores and high-exposure regions were noted in this age range and in children aged 24–61 months, but they did not meet statistical significance [32]. These age distributions were utilized in the second Ecuadorian study [33], which tracked developmental delays by calculating the percentage of children whose scores were two standard deviations below the standardized mean score. This study additionally measured sociodemographic factors. Delayed gross motor skills were displayed in 30.1% of children 3–23 months, while delayed problem-solving and fine motor skills were noted in 73.4% and 28.1% of children aged 48–61 months, respectively. In regards to sociodemographic factors, maternal income and monthly household income was positively correlated with problem-solving and communication abilities [33].

In a separate population of 50 children in Mexico aged 48–62 months, those in a high-exposure region demonstrated decreases in various motor skills-- stamina (*P *= 0.05), fine eye-hand coordination (*P* = 0.009), and gross coordination (*P* = 0.034)-- compared to children living in low exposure settings, and decreases in cognitive abilities reflected by 30-minute memory (*P* = 0.027) and the ability to draw a person (*P* < 0.0001) [44].

#### Questionnaire/Interview.

Self-reported questions regarding pesticide exposure were the primary dependent variables in six studies conducted in Ecuador (n = 2), Tanzania (n = 1), South Africa (n = 1), Colombia (n = 1) and Brazil (n = 1). Five studies were judged as good quality, with one study judged as fair [54].

The Ecuadorian studies utilized children of different ages from the EcoSalud Project population [31,34]. One Ecuadorian study measured exposure levels of children aged 3−23 months based on the nature of their mothers’ work during pregnancy, finding that children whose mothers worked in the flower industry exhibited an 8% decrease in communication scores (95% CI [−16%, 0.5%]) and fine motor skills (13% decrease; CI [−22, −5]), and were more likely to experience poor visual acuity (OR 4.7; CI [1.1, 20]) [31]. The second Ecuadorian study investigated the role of certain behaviors and risk factors with decreased motor and cognitive performance in children 24−61 months old [26]. Specifically, decreases in gross motor (4.2% decrease; 95% CI [−6.3, 1.3]), fine motor (3.5% decrease; 95% CI [−6.9, 2.6]), and problem-solving skills (5.5% decrease; 95% CI [7.5, 1.0]) were reported in children who spent longer amounts of time playing outside. Additionally, playing with irrigation water was correlated with decreased child performance on tests of fine motor (8.2% decrease; 95% CI [9.3, 0.53]), problem solving (7.3% decrease; 95% CI [8.40, 0.39]), and visual motor skills (3.4% decrease; 95% CI [12.00, 1.08]). Interestingly, children with mothers currently working in the flower industry scored significantly better on developmental tests, which may be indicative of the role sociodemographic factors, such as maternal employment, play in development [34].

A study of 286 mother-child pairs in Tanzania also analyzed the effects of both pre- and postnatal exposures on child neurodevelopment [28]. Mothers of children ranging from 0–6 years answered questions pertaining to the children’s and mothers’ exposure to pesticides during pregnancy and throughout their lifetimes. Statistically significant associations (*P* < 0.05) were found between neurodevelopmental delays and multiple variables. When the odds ratio was adjusted for the mother’s age, the home’s distance from a farm [aOR = 9.4 (4.2–20.5), *P* = 0.000] and working while pregnant [aOR = 5.8 (1.29–26.3), *P* = 0.022] remained as statistically significant effects. Additionally, the results suggest that children’s weight status can modify the neurodevelopmental impact of farm-working during pregnancy [aOR = 7.8 (1.29–36.3)] [28].

Children used a questionnaire to self–report behaviors related to pesticide exposures in a South African study of 1001 children between 9–16 years of age [55]. Overall, the results found that pesticide-exposing behaviors were associated with lower cognitive scores, but different behaviors altered distinct neurodevelopmental outcomes. For instance, a negative correlation was specifically noted between pesticide-related farm activities and multi-tasking accuracy scores (β = − 2.74, 95% CI [− 5.19, − 0.29], *P* = 0.03), as well as between eating fruit directly from a vineyard/orchard and motor screening speed (β = − 0.06, 95% CI [− 0.11, − 0.01], *P* = 0.02) and rapid visual processing accuracy scores (β = − 0.02, 95% CI [− 0.03, 0.00], *P *= 0.02). Meanwhile, picking crops from a field was associated with lower strategy in spatial working memory (β = − 0.29, 95% CI [− 0.56, − 0.03], *P* = 0.03) and lower paired associated learning (β = − 0.88, 95% CI [− 1.60, − 0.17], *P* = 0.02) [55].

Furthermore, a study based in Colombia evaluated the prenatal and postnatal pesticide exposure of 232 children between the ages of 7–10 years using an adapted 100-item questionnaire completed by the child’s mother [56]. Using the Wechsler Intelligence Scale for Children, child scores on working memory (exposure at school: β = − 3.46; 95% CI [− 6.65, − 0.28]) and verbal comprehension (exposure at home: β = − 3.38; 95% CI [− 6.97, 0.21]; exposure at school: β = − 3.26; 95% CI [− 6.52, − 0.00]; prenatal exposure: β = − 3.42; 95% CI [− 8.36, 1.51]) were associated with varying methods of pesticide exposure (postnatal at home or at school, or prenatal). Although there was an observed relationship, effects of exposure on processing speed, perceptual reasoning, and full intelligence quotient (IQ) scores were not statistically significant, due to imprecise estimates. Prenatal pesticide exposure only was found to be associated with overall IQ in children (β = − 3.15; 95% CI; − 7.14, 0.84), but there was no relationship with IQ for postnatal exposure at home or at school [56].

Results from the study conducted in Brazil suggest that certain impairments may be associated with rural versus urban living, however their findings did not meet statistical significance (54). Overall, self-reported data from these six studies were mixed, with no clear association between self-reported exposure to pesticides and neurodevelopment.

## Discussion

The purpose of this review was to explore the potential effects of pesticide exposure on neurodevelopment in LMICs to inform protective actions for children’s neurodevelopment. Of the 31 studies in this review, 23 reported significant associations between pesticide exposure and impaired neurocognitive development in at least one domain; three studies reported significant association with impaired behavioral problems. While the data do not provide adequate evidence to implicate a specific pesticide or susceptibility of a specific neurodevelopmental domain, our results do suggest that this is an area that should be further investigated. These results are consistent with existing literature focused primarily on high-income countries [11,12,57,58].

The associations observed between pesticide exposure and neurodevelopment appears to be highly dependent on the method of exposure measurement. Both child- and maternal-level measurements indicate a consistent significant association between pesticide exposure and neurodevelopment with tests of serum measurement of acetylcholinesterase, region of residence, and questionnaires/interviews, but not with child-level urine, maternal serum, and prenatal urine sampling. A possible explanation for the variability within results could be the sensitivity of the diversified measurement methods. One study found that organophosphate levels were more readily detected in sweat than in either blood or urine, and another found moderate reliability within prenatal, intra-individual dialkyl phosphate levels in the urine [59]. A separate study reported that the reliability of urinary analysis varied by pesticide class. Specifically, TCPy did not accurately reflect the application of chlorpyrifos or contact with its residue, while analysis of IMPy and 3-PBA was more sensitive to the application of diazon and pyrethroids [60].

Urine sampling offers unique strengths when measuring recent, acute exposures to rapidly metabolized pesticides exposures, but its varying results represent limitations for capturing the relevant timing of pesticide exposure. For example, in an included study of Thai children living in farming regions, some positive associations were surprisingly shown between exposure to pesticides and motor speed, learning, and memory [45]. Though this relationship was notable, the effect was modest and not consistently observed across domains. This study also reported that TCPy exposure in children was *higher* during low pesticides use season, rather than high pesticides use season [45], supporting the potential impact of timing on pesticide exposure measurements. As such, without being able to measure chronic pesticide exposure, as well as its intensity and cumulative effect, the neurotoxicity profile of pesticides through urine sampling remains incomplete. This may offer implications for the lack of relationship found between neurodevelopment and urine organophosphates in the included articles.

Although not utilized by any of the studies in this review, hair sampling has been found to be an effective method for measuring pesticide levels and the time course of exposure. Hair sampling may detect the parent compounds an individual is exposed to, rather than only the metabolites detected through urine sampling [59,60], coupled with the ability to identify multiple classes of pesticides from each sample [61]. Hair analysis is not without limitations-- exogenous (from dust and other sources) versus endogenous pesticide contamination is not always clear in pesticide measures from hair analysis. Additionally, expensive equipment to conduct hair sampling remains a barrier for LMICs to conduct, potentially explaining its absence in this review. Specific preparation procedures have been tested, but not yet universally established for hair sampling [62]. However, future studies could consider this as a potential methodology.

It is also important to note that sensitivity can be impacted by specificity. Cholinesterase activity, for example, is only reflective of organophosphate and carbamate exposures [63]. While some chemicals can be directly measured with spot urine sampling, others are metabolized too rapidly in the body to be measured directly. In these cases, metabolites are measured instead, as they break down at a stable rate. In some instances, different chemicals break down to the same metabolite, as with 3-PBA, and a secondary measurement method is needed to uncover which substance the subject was exposed to [64]. Thus, if the biological sampling method is too specific, exposures to other pesticides may go unnoticed. Overall, the findings of this review and numerous studies underscore the importance of further considerations on the best methods for appropriate pesticide detection in humans given a study’s specific research objective or design. More clarification about the benefits and challenges to various exposure measurement techniques should be clearly defined so that the interpretation of resulting data can be more assessable.

The finding that urine and serum organophosphates were rarely associated with adverse neurodevelopmental outcomes suggests that some methods of pesticide measurement, being time-specific, may affect study results. Even after pesticide exposures, cholinesterase levels increasingly normalize in the days-to-weeks following the exposure, as the body creates more acetylcholinesterase enzymes [63]. This consideration is not unique to acetylcholinesterase levels; blood and urine sampling methods are also only indicative of recent exposures. The ability of biomonitoring is dually limited by the sensitivity of the test and ability of the body to rid itself of the toxicant. Additionally, half-life must be considered in carbon-containing compounds [65]. One study reported that pesticide exposure levels measured by spot urine sampling varied more within a single child than between groups of children, demonstrating that single measurements are not reliable indicators of one’s long-term exposure to pesticides [66]. Similarly, a review including various analyses of urinary pesticide metabolites concluded that metabolite levels fluctuate greatly over time, which may weaken their indication of true pesticide exposures with smaller sample sizes [60]. Other research regarding the validity of urine sampling indicates a high degree of intra-individual variability of pesticide measures. This highlights the need for measurements at multiple points in time to accurately portray exposure levels [67,68]. Therefore, subjects who are tested beyond a certain window of exposure may appear as though they were never exposed or exposed at a much lower level than is accurate. Any study utilizing biomonitoring is limited by the understanding of temporal exposure considerations for each pesticide class. This is an important area of concern in pesticide exposure research as approximately half of the studies with biological measurements in this review measured intraindividual pesticide exposure more than once.

All nine studies using survey or questionnaire measurements of exposure levels had uniform conclusions of a negative impact on neurodevelopment. Surveys show strength for determining pesticide exposure across the life course; these studies, however, were limited, as there was no direct measure of exposure. Furthermore, surveys and location-based studies are at risk for recall and ecological bias, respectively. The *Improving Exposure Assessment Methodologies for Epidemiological Studies on Pesticides* initiative-- a collaborative effort across the Institute of Occupational Medicine, the Institute of Risk Assessment Sciences at Utrecht University, and the Centre for Occupational and Environmental Health of the University of Manchester-- is attempting to document the impact of recall bias on pesticide exposure classification, while also noting the reliability of various surrogate pesticide exposure measurement methods [69]. Two included studies suggested that recall bias is affected by time and the item being recalled [70,71]. Further results may aid in informing the direction of future studies analyzing the impact of pesticides on human health.

The variability of results between different techniques in this review illustrate that multiple methods of measuring pesticide exposure (i.e., biological markers, region of residence, survey questionnaire) may be applied to understand and quantify the exposure. Researchers must consider the benefits and limitations of each type of pesticide analysis according to the exposure and question of interest. Biomonitoring allows for direct and objective measures of exposure that the subject may not be aware of, but also introduces various challenging factors, including specificity, sensitivity, timing, pathway of exposure, and availability. Non-biomarker methods of pesticide measurement, such as surveys or proximity, provide different opportunities to characterize a participant’s exposures, including the timeframe and sources of exposure. Using region of residence as a method of pesticide quantification can provide information on community-based exposures. These methods, however, do not allow for confirmation of exposure. One review, which investigated various methods of quantifying pesticide exposure, underscored the importance of considering multiple exposure analysis methods [72]. The results recommend utilizing multiple biomarker modalities in future research so that less weight is allotted to indirect measures when policymaking for pesticide exposure prevention in children [72].

Many well-identified risk factors for adverse neurodevelopmental outcomes in LMICs are recognized and should be considered when designing studies in this research area. These include maternal education, parental habits such as smoking, nutrition, socioeconomic status, home environment, and other environmental exposures, such as cadmium and mercury, and are known to influence neurodevelopmental outcomes, which can have significant effects on study results [73–78]. The included articles in this review tended to evaluate nutrition (largely via anthropometric measures) and socio-economic status [28,33,47], which appeared to show positive interaction effects when mitigating the relationship between pesticide exposures and neurodevelopmental outcomes. Notably, in three studies, the nature of prenatal or current maternal employment was closely associated pesticide exposures [28,31,34]. From the data available in these studies, the difference in neurodevelopmental outcomes among children exposed to pesticides through pre- versus postnatal maternal employment could be attributable to the positive benefits associated with maternal employment, such as greater income, rather than solely the exposure itself. This dichotomy between pre- and postnatal maternal employment highlights the complex relationship between sociodemographic factors and the timing of pesticide exposures, as it is impossible to discern whether the difference in neurodevelopmental outcomes resulted from the timing of the pesticide exposure (e.g., children exposed prenatally rather than postnatally are more likely to suffer neurodevelopmental deficits). It is likely that both the timing of exposure and sociodemographic factors impact neurodevelopmental outcomes to some degree, but the question remains: do the positive benefits associated with maternal employment – no matter the industry – outweigh the negative neurodevelopmental impacts associated with maternal pesticide exposure? Answering this question is important in establishing guidelines that will protect children’s neurodevelopment.

This review highlights the limited evidence base derived from LMIC settings that addresses pesticide exposure in the early life course. One major gap is the adequate inclusion of controls in some studies and lack of standardized norming data for many of the study populations. It was specifically noted that this data was not available in Ecuador [33]. Without this data, it is difficult to discern whether the children tested in these studies had neurodevelopmental delays or would differ from unexposed peers that were otherwise similar. This is an inherent problem when conducting studies in LMICs, where less funding is available for creating standardized developmental scales for specific populations.

Another gap appears in infant pesticide exposure analyses. Currently, exposure data for infants is based on either maternal pesticide exposure measurement during gestation or subjective maternal recall. For example, the results of the maternal serum studies in Mexico across varying ages showed that the impact of prenatal pesticide exposure on neurodevelopment could be dependent on the age of the subject and the specific tests used [39–42]. Therefore, these exposure quantifications may not accurately reflect infant pesticide exposure levels, depending on extent of transfer to placenta and/or occurrence in breastmilk, thereby compromising true associations with neurodevelopment. Furthermore, some of the studies did not specify when the maternal pesticide measurements were taken (first trimester, second trimester, etc.). As previously discussed, the timing of pesticide measurements may significantly impact the results. Additionally, changes in DNA transcription and brain development occur more rapidly during gestation than any other time of life [79,80], and neurodevelopmental outcomes appear to be highly dependent on the timing of prenatal stressors [81]. Ideally, both maternal and infant measurements would be taken within documented timeframes to determine infants’ exposure to pesticides with more precision.

There is a notable gap in geographical areas where children were examined for the impacts of pesticide exposure on neurodevelopment. All but five of the studies (conducted in Tanzania, Egypt, and Bangladesh) included in this review were conducted in upper-middle income countries [25–29], and *no research was gathered on children in low-income countries*. As children in low-income countries are at the greatest risk for developmental impacts secondary to pesticide exposures, it is imperative that more research be conducted in these areas. Additionally, most of the research was concentrated in identical or very similar regions of two countries— Ecuador and Mexico. To effectively understand the impacts of pesticide exposure on neurodevelopment, a larger, more diversified body of research is needed.

The current study is limited in that variability within research designs prevented comparative analysis between studies. Ideally, this literature review would have allowed for insights on exposure impacts by pesticide type, age of exposure, age developmental analysis, and the type of developmental domain in question. Unfortunately, no consistent patterns were noted in any of these subgroupings, secondary to large differences in the design protocols such as: (a) study design (cross-sectional vs. cohort), (b) age of subjects, (c) prenatal vs. postnatal measurements, (d) method of measuring exposure, (e) developmental test utilized, (f) type of statistical test utilized, and (g) whether adjustments were made for confounding variables. These distinctions also prevented a meta-analysis from being performed.

To develop policy and practice that protects children from neurodevelopmental deficits, we need well-designed studies that represent the populations of children exposed throughout LMICs. Careful attention to exposure assessment methods and timing of exposure assessment to reflect and inform key research questions, use of well-validated outcome measures conducted by trained data collectors, and inclusion of important covariates are further needed.

## Conclusion

In this review, exposure to organochlorines, carbamates, chlorpyrifos, and fungicides were typically associated with worse outcomes in executive functioning, cognition, motor development, and behavior for children, most commonly in Latin America, particularly Ecuador, and sub-Saharan Africa. However, high variability in our findings reflect inadequate data to discern the impact of specific pesticide types and classes, due to limited study areas and variable validity of exposure assessment. Thus, this review exposes limitations in the current evidence base of LMIC-based studies regarding the neurodevelopmental impact of pesticide exposures. LMICs will benefit from access to robust, reliable exposure assessment methods, such as biomonitoring to understand population-level exposures and trends. In terms of policy and practice, this review provides evidence that national and/or local efforts to mitigate harm from well-recognized pesticide exposures in LMICs are likely to have future benefits for the development, health, and well-being of countless children currently impacted by acute or chronic poisoning linked with pesticides. Future coordination in testing and measurement efforts would advance our knowledge in this field.

## Supporting information

S1 Checklist PRISMA 2020 Checklist. (DOCX)

S1 File Search strategy. (DOCX)

S2 File Full titles from systematic search. (XLSX)

S3 File Quality assessment of included articles. (DOCX)

S4 File Description of studies’ geographic distribution. (DOCX)
