# Supplementary material for: Pesticides and neurodevelopment of children in low and middle-income countries: A systematic review
Source: PLoS One. 2025 Jun 11;20(6):e0324375. doi: 10.1371/journal.pone.0324375 (PMC12157097; doi:10.1371/journal.pone.0324375)
Supplement: S1 File — (DOCX) [file pone.0324375.s002.docx]

**S1:** Search Strategy

Search 1*: (((((((((((((((((("Africa"[Mesh]) OR "Developing Countries"[Mesh]) OR "Poverty"[Mesh]) OR "Rural Population"[Mesh]) OR "South America"[Mesh]) OR (africa)) OR (third world)) OR (south america)) OR (rural)) OR (resource limitation)) OR (resource limit)) OR (resource-limited)) OR (resource constraint)) OR (resource-constrained)) OR (poverty)) OR (low-resource)) OR (low-income)) OR (developing countries)) OR (developing country)

Search 2: ((((((((((((((((((((((((((((((("Air Pollution"[Mesh]) OR "Water Pollution"[Mesh]) OR "Environmental Pollution"[Mesh]) OR "Arsenic"[Mesh]) OR "Environmental Exposure"[Mesh]) OR "Metals, Heavy"[Mesh]) OR "Inhalation Exposure"[Mesh]) OR "Iron"[Mesh]) OR "Lead"[Mesh]) OR "Manganese"[Mesh]) OR "Mercury"[Mesh]) OR (polluted environment)) OR (polluted air)) OR (polluted water)) OR (water pollutant)) OR (water pollution)) OR (polluted)) OR (pollutant)) OR (pollution)) OR (mercury)) OR (manganese)) OR (lead)) OR (iron)) OR (inhalation exposure)) OR (heavy metal)) OR (environmental pollutant)) OR (environmental pollution)) OR (environmental exposure)) OR (chemical)) OR (arsenic)) OR (air pollutant)) OR (air pollution)

Search 3: (((((((((((((((((((((((((((((("Attention"[Mesh]) OR "Behavior"[Mesh]) OR "Child Behavior Disorders"[Mesh]) OR ( "Child Development"[Mesh] OR "Developmental Disabilities"[Mesh] )) OR "Cognition"[Mesh]) OR ( "Executive Function"[Mesh] OR "Neuropsychological Tests"[Mesh] )) OR "Hyperkinesis"[Mesh]) OR "Memory, Long-Term"[Mesh]) OR "Memory, Short-Term"[Mesh]) OR "Risk Assessment"[Mesh]) OR "Verbal Learning"[Mesh]) OR (verbal learning)) OR (risk assessment)) OR (neuropsychological test)) OR (neurodevelopmental)) OR (neurodevelopment)) OR (short term memory)) OR (long term memory)) OR (IQ)) OR (intelligence)) OR (hyperkinesis)) OR (executive functioning)) OR (executive function)) OR (developmental disabilities)) OR (developmental disability)) OR (cognitive)) OR (cognition)) OR (child behavioral disorder)) OR (child development)) OR (behavior)) OR (attention)

Search 4: ((((child) OR (children)) OR (childhood)) OR (adolescent)) OR (("Child"[Mesh]) OR "Adolescent"[Mesh])

Search 5: #1 AND #2 AND #3 AND #4

**Note:** original search criteria was later sorted into three separative reviews. Search terms in grey are not relevant to the current review.
