# Supplementary material for: Pesticides and neurodevelopment of children in low and middle-income countries: A systematic review
Source: PLoS One. 2025 Jun 11;20(6):e0324375. doi: 10.1371/journal.pone.0324375 (PMC12157097; doi:10.1371/journal.pone.0324375)
Supplement: S4 File — (DOCX) [file pone.0324375.s005.docx]

**S4: Figure A.** Geographic distribution of included studies


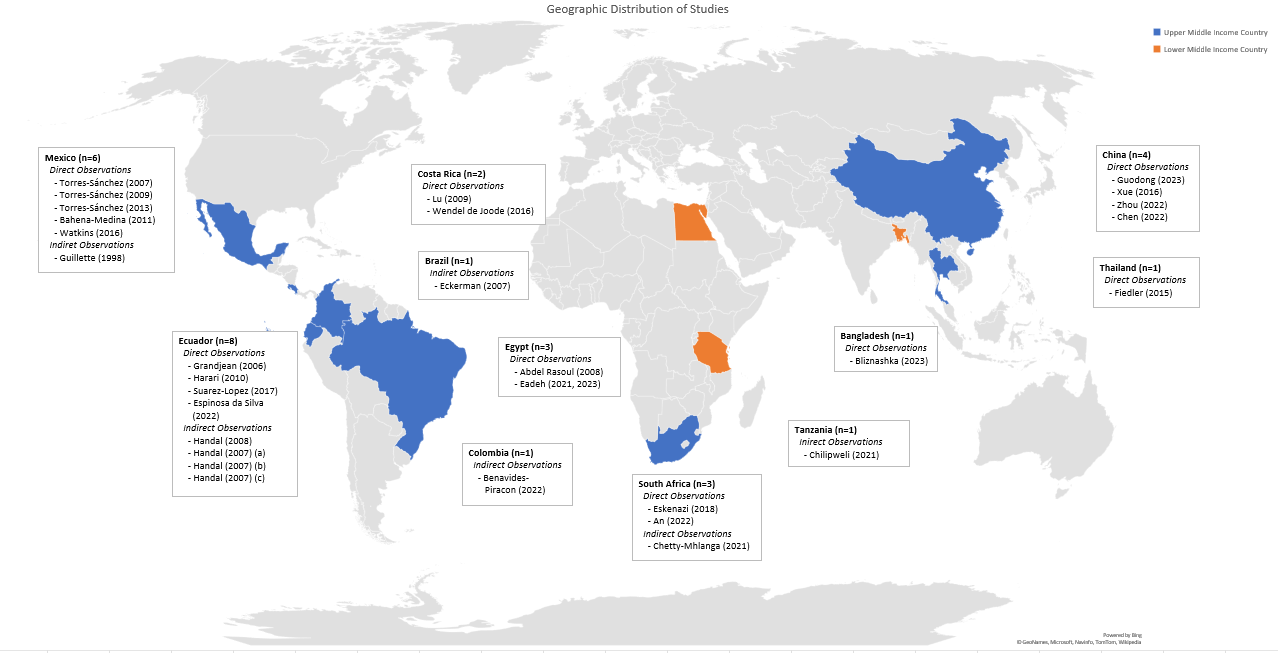


Reprinted from GeoNames and Open Street Map under a CC BY license.

**S4: Table A.** Description of studies’ geographic distribution

| **Region** | **Country** | **Included Studies** | **Measurement** | **Exposure** | **Results across regional studies** |
| --- | --- | --- | --- | --- | --- |
| ***Latin America*** | **Brazil** | *Eckerman* (2007) | Questionnaire (indirect) | Pesticides | There were strong, consistent adverse correlations between pesticide exposure and neurodevelopment, particularly in the youngest participants (ages 10-11) for the majority of cognitive subtests. |
|  | **Colombia** | *Benavides-Piracon* (2022) | Questionnaire (indirect) | Organophosphates; synthetic pyrethroids insecticides; and fungicides | It was found that prenatal and postnatal pesticides exposure adversely affected neurodevelopment in IQ, verbal comprehension, and working memory. |
|  | **Costa Rica** | *Lu* (2009) | Urine (direct) | Organophosphates and pyrethroids (PYR); herbicides (5-chloro-1-isopropyl-3-hydroxytriazole; 2-isopropyl-6-methyl-4-pyrimidinol); 3-Phenoxybenzoic acid (3-PBA); and 3,5,6-trichloro-2-pyridinol (TCPy); 2,4-Dichlorophenoxyacetic acid | Exposure to pesticides showed worse performance on cognitive tests, but there were no significant differences. |
|  |  | *Wendel de Joode* (2016) | Urine (direct) | PYR, mancozeb, TCPy, Chlorpyrifos (CPF), 3-PBA | Greater exposure to TCPy adversely correlated with working memory, visual motor coordination, inattention, and color discrimination. Lower processing speeds and verbal learning scores were associated with 3-PBA and ethylenethiourea, respectively. |
|  | **Ecuador** | *Grandjean* (2006)* | Questionnaire (indirect), acetylcholinesterase and urine (direct) | Pesticides of which organophosphates are the primary | Significant associations were noted with longer reaction time and postnatal organophosphate exposure [P= 0.011]. |
|  |  | *Harari* (2010)* | Questionnaire (indirect), acetylcholinesterase and urine (direct) | Pesticides of which organophosphates are the primary | Prenatal exposure was adversely associated with visual memory, visuospatial performance, and motor skills. |
|  |  | *Suarez-Lopez* (2017) | Time after Mother's Day harvest (indirect), acetylcholinesterase (direct) | Pesticides, insecticides, herbicides, diethyldithiocarbamate fungicides and organophosphate insecticides (primary) | Children examined closer in date to the Mother's Day had worse performance in tests of visuo-spatial processing, sensorimotor skills, and neurobehavior. |
|  |  | *Espinosa da Silva* (2022) | Time after Mother's Day harvest (indirect), acetylcholinesterase (direct) | Organophosphates, insecticides, others possible as not directly measured or noted | Time after harvest had a direct association with scores in attention and visuospatial processing. |
|  |  | *Handal* (2008)* | Questionnaire (indirect) | Organophosphates, carbamates | Prenatal exposure via maternal employment and use of pesticides at home was adversely associated with communication scores, motor skills, and visual acuity. |
|  |  | *Handal* (2007) (a)* | Questionnaire (indirect) | Organophosphates, carbamates | Pesticide exposure via maternal employment was significantly correlated with higher developmental scores. |
|  |  | *Handal* (2007) (b)* | Community of residence (indirect) | Organophosphates, carbamates | Exposure to pesticides showed worse performance in motor and problem-solving skills. |
|  |  | *Handal* (2007) (c)* | Community of residence (indirect) | Organophosphates, carbamates | Living in a high-exposure area was significantly correlated with lower gross motor and socio-individual scores. |
|  | **Mexico** | *Torres-Sánchez* (2007)* | Maternal serum (direct) | Dichloro-diphenyl-dichloroethylene (DDE) | No correlations between DDE and neurodevelopment were found, except for DDE exposure in the first trimester of pregnancy and psychomotor scores [P=0.02]. |
|  |  | *Torres-Sánchez* (2009)* | Maternal serum (direct) | DDE | There were no significant correlations between DDE and neurodevelopment at 12 months of age. |
|  |  | *Torres-Sánchez* (2013)* | Maternal serum (direct) | DDE | There were significant reductions in cognition, quantitative skills, verbal skills, and memory with each increase in unit of DDE. |
|  |  | *Bahena-Medina* (2011)* | Maternal serum (direct) | DDE | Exposure to DDE showed worse performance on cognitive tests of psychomotor and mental development, but there were no significant differences. |
|  |  | *Watkins* (2016) | Maternal urine (direct) | PYR | No correlations between PYR and neurodevelopment were found. |
|  |  | *Guillette* (1998) | Region of residence (indirect) | Organophosphates, organochlorines, pyrethroids | Pesticide exposure was adversely associated with motor development and memory. |
| ***North Africa*** | **Egypt** | *Abdel Rasoul* (2008) | Questionnaire (indirect), acetylcholinesterase (direct) | Organophosphates (various forms of CPF), PYR (or less potent carboxylate) | There was a dose-response effect between pesticide exposure and 3-6 subtests of cognitive deficits. |
|  |  | *Eadeh* (2021)* | Urine (direct) | CPF, TCPy | Mean TCPy was adversely associated with cognitive sub-tests. |
|  |  | *Eadeh* (2023)* | Urine (direct) | CPF, PYR, alpha-cypermethrin, lambda-cyhalothrin, TCPy, 3-PBA, 2,2-dichlorovinyl-2,2-dimethyl-1-cyclopropane carboxylic acid (DCCA) | cis-DCCA was adversely associated with one sub-test related to inattention. |
| ***Sub-Saharan Africa*** | **South Africa** | *Eskenazi* (2018)* | Maternal blood and urine (direct) | Dichloro-diphenyl-trichloroethane (DDT), DDE, PYR | There was a dose-response effect with reduced social-emotional performance for: trans-DCCA, cis-DCCA, and 3-PBA. |
|  |  | *An* (2022)* | Urine (direct) | PYR; 3-PBA; DDE, DDT; DCCA | There was a dose-response effect with behavioral scores in multiple sub-tests for: DDT, DDE, 3-PBA, and cis-DCCA. |
|  |  | *Chetty-Mhlanga* (2021) | Questionnaire (indirect) | Organophosphates, organochlorines, others possible as not directly measured or noted | There was a dose-response effect when engaging behaviors that increased the risk of pesticide exposure and the risk of lower neurodevelopmental outcomes. |
|  | **Tanzania** | *Chilipweli* (2021) | Questionnaire (indirect) | Organophosphates, PYR, carbamates, glycine derivative, phthalic acid diamide, insecticides | There was a dose-response effect between engaging behaviors prenatally and postnatally that increased the risk of pesticide exposure and the risk of lower neurodevelopmental outcomes. |
| ***South Asia*** | **Bangladesh** | *Bliznashka* (2023) | Urine (direct) | 2,4-Dichlorophenoxyacetic acid; TCPy; 4-nitrophenol; malathion dicarboxylic acid; 2-isopropyl-4-methyl-6-hydro-xypyrimidine (IMPy); 4-fluoro-3-PBA; 3-PBA; trans-DCCA | No correlations between pesticide exposure and neurodevelopment were found, except in an inverse association between TCPy and IMPy with motor and cognitive development among the cohort study component. |
| ***East Asia*** | **China** | *Guodong* (2012) | Urine (direct) | Organophosphates | No correlations between organophosphate levels and neurodevelopment were found. |
|  |  | *Xue* (2016) | Urine (direct) | Synthetic PYR pesticides | There was a significant association between PYR exposure and worse mental development. |
|  |  | *Zhou* (2022) | Urine (direct) | CPF | There was a significant direct association between CPF and greater risk of inattention. |
|  |  | *Chen* (2022) | Urine (direct) | PYR; DCCA; 3-PBA | There was a significant association between PYR with DCCA and 3-PBA with worse language and communication scores. |
|  | **Thailand** | *Fiedler* (2015) | Urinary metabolites (direct) | Organophosphates, PYR, CPF, TCPy, 3-PBA, DCCA | No correlations between pesticide exposure and neurodevelopment were found, except for better performance in memory and motor tests among children exposed to DCCA and 3-PBA, though this was not significant. |

Abbreviations: PYR: pyrethroids; 3-PBA: 3-Phenoxybenzoic acid; TCPy: 3,5,6-trichloro-2-pyridinol; CPF: Chlorpyrifos; DDE: dichloro-diphenyl-dichloroethylene; DCCA: 2,2-(dichloro)-2-dimethylvinylcyclopropane carboxylic acid; DDT: dichloro-diphenyl-trichloroethane; IMPy: 2-isopropyl-4-methyl-6-hydroxypyrimidine

*These studies featured the same population, general study design, and overall results, but differed in the way they analyzed and reported data.
